# Using the Oxford Cognitive Screen to Detect Cognitive Impairment in Stroke Patients: A Comparison with the Mini-Mental State Examination

**DOI:** 10.3389/fneur.2018.00101

**Published:** 2018-02-28

**Authors:** Mauro Mancuso, Nele Demeyere, Laura Abbruzzese, Alessio Damora, Valentina Varalta, Fabio Pirrotta, Gabriella Antonucci, Alessandro Matano, Marina Caputo, Maria Giovanna Caruso, Giovanna Teresa Pontiggia, Michela Coccia, Irene Ciancarelli, Pierluigi Zoccolotti, The Italian OCS Group, Chiara Beni

**Affiliations:** ^1^Tuscany Rehabilitation Clinic, Montevarchi, Italy; ^2^Cognitive Neuropsychology Centre, Department of Experimental Psychology, University of Oxford, Oxford, United Kingdom; ^3^Neuromotor and Cognitive Rehabilitation Research Center, Department of Neurological, Biomedical and Movement Sciences, University of Verona, Verona, Italy; ^4^Department of Psychology, Sapienza University of Rome, Rome, Italy; ^5^Neuropsychology Center, IRCCS Santa Lucia Foundation, Rome, Italy; ^6^Neurological Rehabilitation Unit, Auxilium Vitae Volterra, Volterra, Italy; ^7^Physical and Rehabilitation Medicine, Department of Medical and Surgical Sciences “Magna Graecia” University, Catanzaro, Italy; ^8^Neuropsychology Unit, National Health Service, Bari, Italy; ^9^Department of Neuroscience, Neurorehabilitation Clinic, Azienda Ospedaliero–Universitaria Ospedali Riuniti, Ancona, Italy; ^10^Department of Life, Health and Environmental Sciences, University of L’Aquila, Nova Salus s.r.l., L’Aquila, Italy

**Keywords:** stroke, cognitive assessment, Oxford Cognitive Screen, Mini-Mental State Examination, cognitive screening

## Abstract

**Background:**

The Oxford Cognitive Screen (OCS) was recently developed with the aim of describing the cognitive deficits after stroke. The scale consists of 10 tasks encompassing five cognitive domains: attention and executive function, language, memory, number processing, and praxis. OCS was devised to be inclusive and un-confounded by aphasia and neglect. As such, it may have a greater potential to be informative on stroke cognitive deficits of widely used instruments, such as the Mini-Mental State Examination (MMSE) or the Montreal Cognitive Assessment, which were originally devised for demented patients.

**Objective:**

The present study compared the OCS with the MMSE with regards to their ability to detect cognitive impairments post-stroke. We further aimed to examine performance on the OCS as a function of subtypes of cerebral infarction and clinical severity.

**Methods:**

325 first stroke patients were consecutively enrolled in the study over a 9-month period. The OCS and MMSE, as well as the Bamford classification and NIHSS, were given according to standard procedures.

**Results:**

About a third of patients (35.3%) had a performance lower than the cutoff (<22) on the MMSE, whereas 91.6% were impaired in at least one OCS domain, indicating higher incidences of impairment for the OCS. More than 80% of patients showed an impairment in two or more cognitive domains of the OCS. Using the MMSE as a standard of clinical practice, the comparative sensitivity of OCS was 100%. Out of the 208 patients with normal MMSE performance 180 showed impaired performance in at least one domain of the OCS. The discrepancy between OCS and MMSE was particularly strong for patients with milder strokes. As for subtypes of cerebral infarction, fewer patients demonstrated widespread impairments in the OCS in the Posterior Circulation Infarcts category than in the other categories.

**Conclusion:**

Overall, the results showed a much higher incidence of cognitive impairment with the OCS than with the MMSE and demonstrated no false negatives for OCS vs MMSE. It is concluded that OCS is a sensitive screen tool for cognitive deficits after stroke. In particular, the OCS detects high incidences of stroke-specific cognitive impairments, not detected by the MMSE, demonstrating the importance of cognitive profiling.

## Introduction

Stroke is the second most common cause of death and the third most common source of disability (1, 2). Its prevalence and incidence increases with age representing the leading cause of disability in the elderly (2). Patients with stroke have cognitive deficits in a very high proportion of cases [e.g., Ref. (1)], although variables estimates are reported. The differences are likely due to variability in sample characteristics, assessment methods, definitions of impairment, and time interval since stroke onset (1). As cognitive assessment is time consuming, physicians often use smart tools to assess cognitive impairment in stroke survivors that need little time but are often of limited use to highlight cognitive dysfunction, typically yielding relatively low-prevalence rates, below 25% (3, 4). On the opposite, more detailed neuropsychological assessments of domain-specific cognitive impairments consume more time but are better at detecting cognitive impairment, highlighting higher occurrences, ranging from 35 to 92% (5–8). Language, spatial attention, memory, praxis, executive function, and speed of processing are the main impaired cognitive domains (5). Moreover, psychiatric comorbidities such as depression and delirium often occur after a stroke (9). Cognitive deficits interfere with rehabilitation and represent a negative prognostic factor (10, 11), impacting on activities of daily living, quality of life, and return to work (12).

Stroke guidelines recommend the importance of early cognitive diagnosis in order to plan tailored rehabilitation programs (13). Tools, such as the Mini-Mental State Examination [MMSE; (14)] and the Montreal Cognitive Assessment [MoCA; (15)], are widely used as a practical solution to briefly assess cognition post-stroke. However, these instruments were devised for evaluation of patients with dementia and only provide a “domain-general” cognitive score with a single cutoff for impairment. The present study describes the use of a newly devised instrument, the Oxford Cognitive Screen (OCS), against one of these two reference tools, namely the MMSE; in a parallel study, we examined the effectiveness of the OCS against the MoCA (16).

Interest in using the MMSE as a comparison chiefly stems from its wide use; indeed, it is one of the most widely tests used in clinical practice. Early reviews emphasized the reliability and construct validity of the MMSE to capture moderate-to-severe cognitive impairment (17). However, the limits of the MMSE are also well-known particularly in the assessment of stroke patients (18, 19). In spite of this, the MMSE is still one of the instruments which is most widely used nowadays in clinical settings to detect global cognitive impairment in patients with stroke (20–30). In particular, it is used as a diagnostic index in the stroke units to plan the rehabilitation interventions as well as in the identification of cognitive profiles after non-dementia cerebro-vascular events (21, 29, 31–33).

The key problem in using the MMSE to assess stroke sequelae is that it does not explicitly assess common post-stroke domain-specific impairments such as neglect, executive function, apraxia, and aphasia. Indeed, performance on the MMSE can be confounded by co-occurring difficulties in these domains. For example, a patient with expressive aphasia will maximally score 4 points (out of a maximum 30) as the large majority of tasks require spoken language. This would lead to a potential misclassification of patients as “dementia” where there is a specific language impairment. Similarly, specific cognitive impairments may be “missed” in patients with stroke. This lack of specificity contrasts the indications of several clinical guidelines which emphasize the need to assess performance across different domains of cognition after stroke [e.g., Ref. (2, 34)].

The OCS was recently developed with the specific aim of describing the cognitive deficits after stroke (35); OCS was devised to be inclusive and un-confounded by aphasia and neglect. It can be administered within 15 min, can be delivered at the bedside, is easy to administer and score, can be used in relatively acute phase (after 3 days from onset) and provides a “snapshot” of a patient’s cognitive profile useful to define the rehabilitative program. The possibility to have separate cutoff for each of the tasks used allows obtaining a cognitive pinpointing strengths and weaknesses of individual stroke patients.

The scale consists of 10 tasks encompassing five cognitive domains: attention and executive function, language, memory, number processing, and praxis. Furthermore, it includes a brief evaluation of visual field defects. Administration is simple and brief (ca. 15 min) making it also suitable for immobilized patients. Demeyere et al. (16) provided initial data on a sample of stroke patients indicating the ability of the scale to detect differentiated profiles across the various domains and also reported a greater sensitivity of OCS over MoCA.

In order to assess whether this new instrument provides a sensitive and practical first line assessment supporting wider adoption, the present study aimed to compare the OCS with the MMSE in detecting cognitive symptoms after stroke, thereby providing further data on the sensitivity and specificity of the OCS in the identification of cognitive deficits in a relatively large sample of first stroke patients. We also examined OCS performance as a function of subtypes of cerebral infarction [based on the Bamford classification; (36)] and clinical severity [based on the National Institutes of Health Stroke Scale, NIHSS; (37)].

## Materials and Methods

### Patients

Fourteen different Italian rehabilitative centers participated in this study. In each center, the patients were consecutively recruited from February to October 2016 and selected based on the following inclusion and exclusion criteria.

– Inclusion criteria: Patients at first episode; both gender; aged from 18 to 90 years; distance from stroke onset within180 days; patients who were able to give informed consent; ability to concentrate for a minimum of 20 min (as judged by the care team).
– Exclusion criteria: Patients with a previous stroke; the presence of premorbid psychiatric or neurological disease; distance from onset <72 h; patients unable to give informed consent; patients without ability to concentrate for <20 min (as judged by the care team).

The sample dimension was calculated through a power analysis carried out with the G*Power 3.1 program (finale sample tested *n* = 325 patients; see below for a description).

The research was approved by the Tuscany-South East Vast Area Ethical Committee (n. 376/2015); all patients signed the informed consent.

### Cognitive Screening Tests

The OCS (35) assesses five cognitive domains: attention and executive function, language, memory, number processing, and praxis. Furthermore, visual field deficits are also assessed.

A brief description of tests according to each domain follows (in brackets the order of sub-test presentation):

- Language domain:
  ◦ Picture naming (1): The patient is requested to name four pictures in order to assess the expressive language.
  ◦ Semantics (2): The patient has to point the picture asked, choosing between four pictures simultaneously shown.
  ◦ Sentence reading (5): One centrally aligned 15-word long sentence is presented and the participant is asked to read it aloud. The sentence is later used for an unanticipated verbal memory task.
- Memory domain:
  ◦ Orientation (3): The participant is asked about which city is in, the time of the day, the month, and the year.
  ◦ Recall and recognition (10): The patient is asked to recall the sentence previously read in the “sentence reading task.” If the patient is unable to recall the sentence, a page with four options for each irregular word is shown. A further four multiple-choice questions are then given to address non-verbal, episodic memory through task recall.
- Number processing domain:
  ◦ Number writing (6): The patient is asked to write down the numbers heard.
  ◦ Calculation task (7): The patient is required to make two additions and two subtractions.
- Attention and executive function domain:
  ◦ Broken hearts (8): A page with 150 semi-randomly positioned hearts (50 full hearts and 50 broken on the left and 50 on the right) is presented. The task is to cancel out all complete hearts while not crossing out hearts broken on the left or on the right with a 3-min time limit.
  ◦ Trails task (11): Stimuli are pages with circles and squares of different sizes. The two baseline tasks require drawing a line between either circles or squares going down in size and alternating between circles and squares, again going down in size (largest square to largest circle to next largest square, etc.).
- Praxis domain:
  ◦ Imitating meaningless gestures (9): This test requires to patient to mirror imitate, with their better hand, a series of meaningless hand and finger actions made by the examiner.
- Visual field (4): The examiner faces the participant and raises both hands, first to the participant’s upper visual fields, and then to the lower fields asking to wiggle the fingers of the left or right hand.

The OCS can be administered in about 15 min and can be delivered at the bedside, whenever necessary. The Italian version of the scale, with normative data on a sample of 20- to 80-year-old individuals, was used (38).

The MMSE is a widely used tool for cognitive assessment. As a domain-general cognitive screen, the MMSE ultimately returns a pass/fail judgment, based on a single overall score. The instrument can be divided into two main sections: the first requires only verbal feedback and includes the evaluation of orientation, memory, and attention. The second part assesses the naming skills, to follow verbal and written commands, write spontaneously a sentence, and copy a complex polygon. Although the MMSE contains subsections, there are no sub-domain cutoffs. The total maximum score is 30 and the total time of administration is about 10 min. Normative values, adjusted for age and education, are available for the Italian elderly population and the cutoff <22 was established for pathological performance (14).

The OCS and MMSE were presented on the same day; order of presentation of the two tests was counterbalanced on an ABAB basis for each rehabilitation center. Tests were administered in a quiet and comfortable setting.

### Clinical Scales

The NIHSS was designed to evaluate stroke severity. It consists of 11 items, the total score ranging from a minimum of 0 (normal neurological functioning) to a maximum of 42 (severe neurological damage). The following categories are considered: minor stroke = 1–4, moderate = 5–15, moderate to severe = 16–20, severe = 21–42. The time of administration is about 5/8 min (37).

The Bamford Classification (36) allows clustering patients with cerebral infarction according to some distinctive features, placing them into four groups: Lacunar Infarcts, Total Anterior Circulation Infarcts, Partial Anterior Circulation Infarcts, and Posterior Circulation Infarcts (POCI) on the basis of the signs and symptoms.

### Statistics

For descriptive purposes, we examined the percentage (%) of patients to whom the OCS could not be administered, as well as the % of patients falling in one or more domains of the scale.

In order to examine the significance of the association between different kinds of classification, we used Fisher’s exact test for categorical data when expected values in at least one cell was below 5. This was the case for the Bamford classification, OCS, and MMSE. Conversely, χ^2^ tests were used to examine contingency tables with expected values always above 5, as in the case of NIHSS values.

## Results

Over the 9 months of data collection, we recruited a total of 325 patients (178 males and 147 females). The mean age was 69.46 years (SD = 12.53) while years of education averaged 9.07 years (SD = 4.52). The average time from stroke was 33.9 days (SD = 41. 8). Demographic and clinical features of the sample are given in Table 1. Based on CT scan data 278 patients had ischemic and 44 hemorrhagic stroke etiologies. Unilateral lesions were found in 306 patients: 122 had left-hemisphere damage (LHD) and 184 right-hemisphere damage (RHD). Only 19 patients had bilateral (midline) or cerebellar lesions.

**Table 1:** Characteristics of the sample.

	Category	No. of patients (325)	%
Gender	Male	178	54.7
	Female	147	45.2

Etiology	Ischemic	278	85.5
	Hemorrhagic	44	13.5

Lesion lateralization	Unilateral left hemisphere	122	37.5
	Unilateral right hemisphere	184	56.6
	Bilateral/cerebellar	19	5.8

Vascular territory affected for ischemic patients:Bamford classification(*n* = 274)	TACI	58	17.8
	LACI	76	23.3
	PACI	91	28
	POCI	54	16.6

Stroke severity:NIHSS	Minor	171	52.6
	Moderate	136	41.8
	Moderate to severe	13	4
	Severe	4	1.2

The OCS proved generally easy to administer and only for three patients (0.9%) the scale could not be given at all. In a few cases, some tests could not be administered (see Table 2); the main reasons for this were severe visual or motor impairment, fatigue or inability to understand the instructions due to severe global aphasia. The MMSE could not be administered to six patients.

**Table 2:** Number and percentage of patients for whom Oxford Cognitive Screen tests could not be administered.

Domain	Tasks	*n*	%
Language	Picture naming	4	1.2
	Semantics	3	0.9
	Sentence reading	5	1.2

Memory	Orientation	3	0.9
	Recall and recognition	3	0.9
	Episodic memory	3	0.9

Number	Number writing	5	1.5
	Calculation	3	0.9

Perception	Visual field	5	1.5

Spatial attention	Hearts cancelation	17	5.2
	Space asymmetry	21	6.4
	Object asymmetry	21	6.4

Praxis	Imitation	5	1.5

Executive function	Baseline score	28	8.6
	Shifting score	26	8

About a third of patients (*n* = 115; 35.3%) had a performance lower than the cutoff (<22) (14) on the MMSE, whereas 91.6% (*n* = 295) were impaired in at least one OCS domain (Table 3), indicating higher incidences of impairment for the OCS than MMSE (Fisher’s exact *p* < 0.0001). More than 80% of patients (*n* = 263; 81.6%) showed an impairment in two or more cognitive domains of the OCS. Using the MMSE as a standard of clinical practice, the comparative sensitivity of OCS was 100%, indicating that no patients had an impairment in MMSE but not in OCS. Infact, 180 patients with cognitive deficit were detected as positive to OCS but not to MMSE, indicating that OCS did not show false negative.

**Table 3:** Number (and percentage) of patients who passed the cutoff for Mini-Mental State Examination (*n* = 208) but failed in one or more of the Oxford Cognitive Screen tests.

Domain	Task	*n*	%
Language	Picture naming	71	34.1
	Semantics	10	4.8
	Sentence reading	71	34.1

Memory	Orientation	17	8.1
	Recall and recognition	61	29.3
	Episodic memory	65	31.2

Number	Number writing	35	16.8
	Calculation	76	36.5

Perception	Visual field	23	11

Spatial attention	Hearts cancelation	95	45.6
	Space asymmetry	43	20.6
	Object asymmetry	14	6.7

Praxis	Imitation	33	15.8
Executive function	Baseline score	77	37
	Shifting score	65	31.2

Out of the 208 patients with normal MMSE performance (i.e., a score of at least 22), 180 showed impaired performance in at least one domain of the OCS (Table 3). Note the high proportion of patients (about one-third or more) with impaired performance in picture naming and sentence reading (language domain), calculation (number processing domain), recall and recognition and episodic memory tests (memory), and hearts cancelation (attention domain). Only 27 patients were not impaired in both cognitive scales.

**Figure 1:**
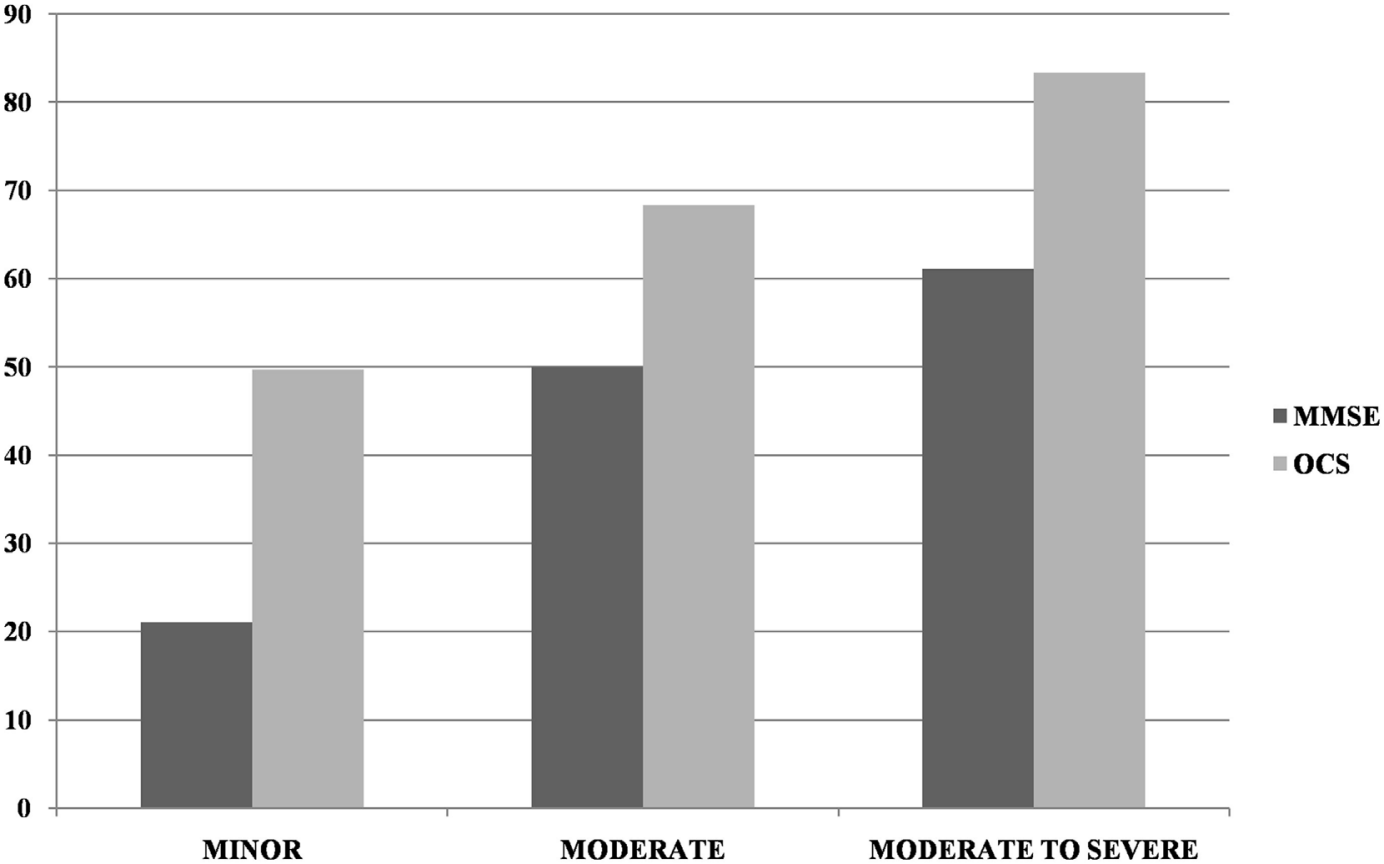
Incidence of impairment at Mini-Mental State Examination (MMSE) and Oxford Cognitive Screen (OCS) (at least one domain) as a function of NIHSS severity (minor = NIHSS 1–4, moderate = 5–15, moderate-severe = 16–20). Too few patients were in the severe NIHSS category to allow for reliable comparisons.

Most patients (*n* = 266; 82.1%) had a motor, sensory, or cognitive deficit on the NIHSS (proportion of patients as a function of stroke severity is presented in Table 1). There was a weak, but significant, inverse correlation between the severity of the NIHSS and MMSE corrected score (*r* = −0.186, *p* = 0.001). Incidence of impairment on the OCS was significantly higher than that in the MMSE in the minor (χ^2^_(1)_ = 41.18, *p* < 0.0001) and moderate (χ^2^_(1)_ = 32.82, *p* < 0.0001) categories but not in the moderate-severe category (χ^2^_(1)_ = 1.16, *p* = 0.28; see Figure 1).

**Figure 2:**
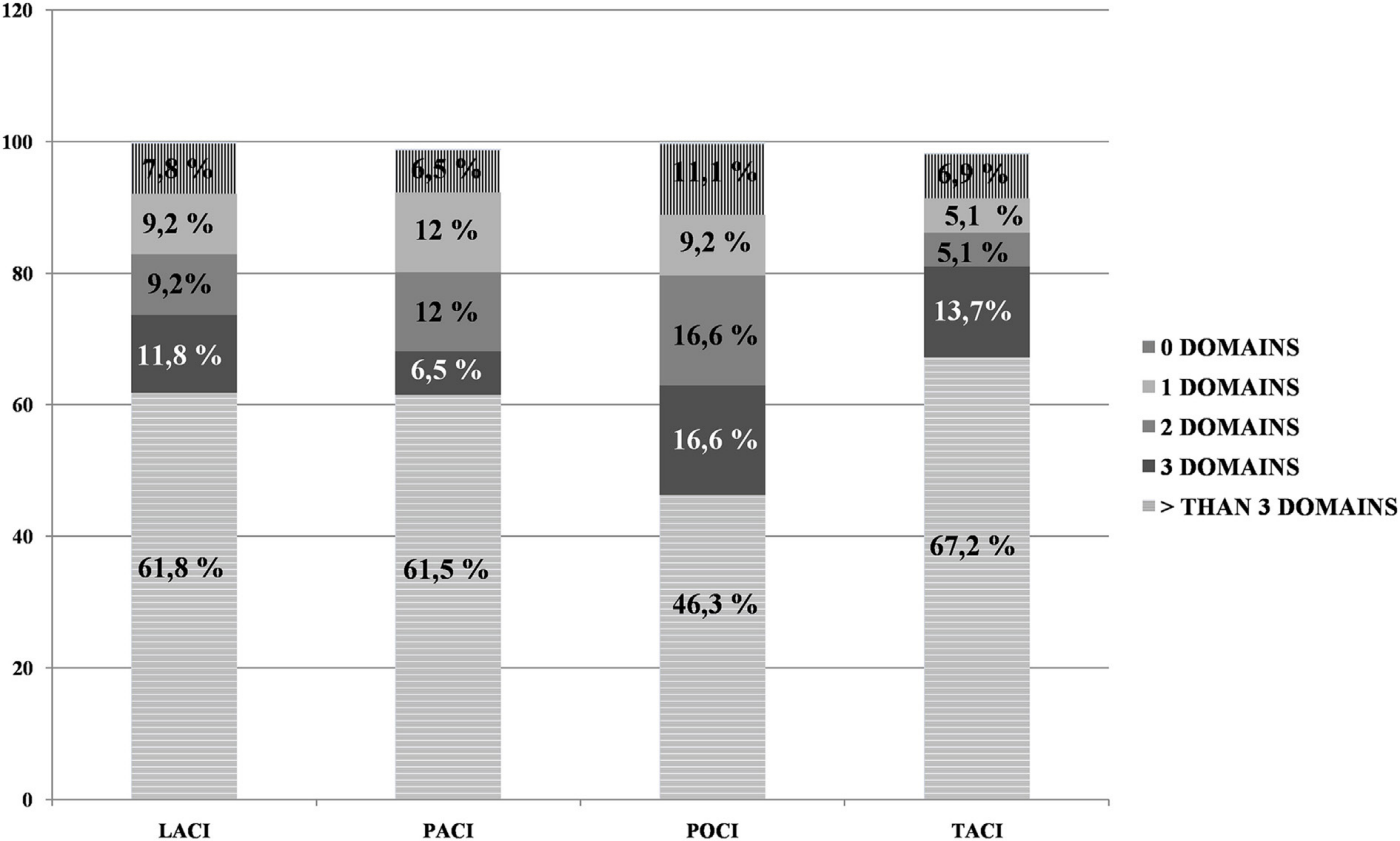
Percentage of Oxford Cognitive Screen domain impairments for each of the four Bamford categories.

In relationship to the Bamford Classification, the frequency of impairments in OCS domains was higher than the MMSE impairments for all categories (all Fisher’s exact tests at least *p* < 0.01). When considering the number of domain impairments on OCS, fewer patients demonstrated widespread impairments (i.e., more than three domains) in the POCI category than in the other categories (see Figure 2).

With regards to lesion side, there were 37.5% (*n* = 122) patients with LHD and 56.6% (*n* = 184) patients with RHD. The remaining patients had bilateral and/or cerebellar damage. Among LHD patients, 43.4% were deficient in the MMSE, while 91.8% were impaired in at least one OCS domain. Among RHD patients, 32% were deficient in the MMSE, while 90.2% were impaired in at least one OCS domain.

Incidence of impairments in the MMSE and in the different OCS domains/tests is reported in Table 4 separately for the overall sample, the LHD and RHD patients. LHD patients had higher frequency of impairments in the language domain (sentence reading test) and in the memory domain (recall and recognition test). In the broken hearts test, a higher percentage of RHD patients showed inattention for left space while a higher percentage of LHD patients showed a higher percentage of right inattention for the right space. Finally, there was a trend for RHD patients to be more impaired in the shifting score (executive function domain). As for the MMSE, RHD patients were more frequently impaired than LHD patients.

**Table 4:** Percentage of patients obtaining a pathological score in the Mini-Mental State Examination (MMSE) and Oxford Cognitive Screen (OCS) tests as a function of lesion lateralization.

Screen	Cognitive domain	Task	Lateralization			
			Overall (%), *n* = 325	Unilateral left (%), *n* = 122	Unilateral right (%), *n* = 184	*p*-Value
MMSE	Overall score	Cutoff = 22	35.3	43.4	32	0.039

OCS	Language	Picture naming	43.6	47.5	40.7	0.156
		Semantics	11.08	10.6	12.5	0.72
		Sentence reading	49.8	57.3	45.1	0.019
	Memory	Orientation	21.8	22.1	22.8	1.000
		Recall and recognition	47.0	56.5	42.9	0.013
		Episodic memory	48.3	48.3	47.2	0.725
	Number	Number writing	36	40.1	34.2	0.223
		Calculation	50.7	55.7	48.3	0.158
	Perception	Visual field	15.6	11.4	18.4	0.147
	Spatial attention	Hearts cancelation	55.6	50	60.3	0.121
		Space asymmetry	31.3	28.6	35.3	0.316
		Left inattention > 3	20	20.4	26.09	0.000
		Right inattention < *−*3	14	12.3	10.3	0.018
		Object asymmetry	6.7	7.3	4.8	0.456
		Left inattention > 2	10.1	7.3	12.5	0.122
		Right inattention < −2	6.7	6.5	4.8	0.332
	Praxis	Imitation	28.3	29.5	27.7	0.697
	Executive function	Baseline score	34.7	36.07	34.2	0.71
		Shifting score	32.3	25.4	36.9	0.076

## Discussion

Our study examined the detection of cognitive symptoms after a first stroke by comparing two scales, the widely used MMSE, and a new instrument, the OCS. The results showed a much higher incidence of cognitive impairment with the OCS (91% at least one domain impairment) than with the MMSE (35%), and demonstrated no false negatives for OCS vs MMSE (giving OCS a comparative sensitivity of 100%). These data support recent reports on the under-detection of post-stroke cognitive impairments of MMSE (18, 39) and further highlight the shortcomings of using tests designed for dementia (16, 40) within a stroke population. In particular, in a parallel study we demonstrated the greater sensitivity of the OCS scale over the MoCA scale, another instrument originally devised for the detection of dementia symptoms. In this respect, it may be noted that there is some indication of a greater sensitivity of MoCA over the MMSE (20, 39, 41); however, there is also counter-evidence pointing to a substantial equivalence between these two instruments [(42); for a review see Ref. (43)]. This is particularly the case when cutoffs adjusted for age and schooling (21) are used (22) as in the present study. Overall, it appears that cognitive deficits after stroke are detected more effectively by a tool (OCS) selectively devised to capture deficits after stroke than by tools originally devised for dementia, such as MMSE and MoCA.

The detection of discrepancy between OCS and MMSE was particularly strong for patients with milder stroke, i.e., in the lower NIHSS range. For patients in the moderate-to-severe category, there was no longer a difference between the cognitive screens, as almost all patients had severe cognitive impairments, likely to be detected by any type of cognitive screening. This finding is in keeping with previous observations that MMSE is not sensitive to mild cognitive deficits [e.g., (44)].

Over 80% of patients showed an impairment in more than one cognitive domain on the OCS, demonstrating the importance of cognitive profiling (45). A recent study highlighted that the MMSE was only able to detect much lower frequencies of cognitive dysfunction [24–50% and 6–31%, respectively; (1)]. The OCS detected high incidences of stroke-specific cognitive impairments, not detected by the MMSE: e.g., neglect was present in 31% of the patients unimpaired on the MMSE. Similarly, 49% of patients failed in the sentence reading task (Table 4). These stroke-specific problems are not detected in the MMSE as they are considered to be unimpaired within a dementia profile; so, they are not assessed in this screen. Given that such stroke-specific deficits may be targeted by specific interventions (e.g., occupational therapy for apraxia, scanning or prism therapies for neglect, specific communication therapies for different language impairments, etc.), domain-level cognitive profiling is likely to be effective for planning individually tailored interventions.

Considering the Bamford classification, fewer patients presented widespread cognitive impairments (i.e., more than three OCS domains) in the POCI category. It is known that POCI stroke infarcts do not affect subcortical structures involving the larger attention networks. This is in line with other studies that indicate that patients with lesions in the vertebro-basilar territory have clinical features that make them different from patients with lesions in the cortical or subcortical areas (46). Infact, the impairment of these posterior areas most frequently cause motor deficits, articulatory speech difficulties, dysphagia, vertigo, nausea or vomiting, facial palsies, or lower cranial nerve deficits (46). In contrast, strokes in these posterior areas result in less disruption of brain regions involved in cognition (47). Thus, patients with ischemia in these areas typically show better cognitive outcomes and cognitive deficits, such as aphasia and neglect, are infrequent (47).

Finally, some features of the OCS scale that indicate its feasibility for clinical use on post-stroke patients should be emphasized. The scale allows detailed screening of the cognitive domains that are compromised following stroke, through the separate assessment of memory, language, number cognition, praxis, executive functions, and attention; visualization of patient’s strengths and weaknesses is made possible through reference to separate cutoffs for single subtests. Administration is compatible with the presence of severe language impairments as OCS includes items that do not require language-based answers (e.g., the patient has to indicate the answer among different visual alternatives); similarly, the influence of left unilateral spatial neglect is minimized by arranging targets vertically whenever appropriate. Finally, the administration is generally short (usually within 15 min) and well suited to daily clinical practice, including the patient’s bedside. The experience with patients in the present study confirmed these general comments. Only for three patients (i.e., less than 1%) we could not administer the OCS; in a limited number of cases, individual subtests could not be given (this ranged from 0.9 to 8.6% depending on the sub-test).

### Conclusion

Results indicate that OCS presents a sensitive screening procedure for cognitive deficits after stroke. In particular, the OCS detects high incidences of stroke-specific cognitive impairments, not detected by the MMSE, demonstrating the importance of cognitive profiling. In view of the wide use of MMSE, we propose that this conclusion calls for a substantial revision of the clinical standards for the screening procedures of cognitive deficits after stroke.

## Italian OCS Group

**Chiara Beni**, **Fabio Giovannelli** (Arezzo); **Ivana Bureca** (Rome); **Mauro Zampolini**, **Adonella Benedetti** (Perugia); **Nicola Smania**, **Cristina Fonte**, **Elisa Ghirardi** (Verona); **Maurizio Iocco** (Catanzaro); **Federica Galli** (Ancona); **Laura Prospero**, **Adriana Gadaleta**, **Maristella Scattaglia**, **Franco Valluzzi** (Bari); **Nicoletta Caputi** (L’Aquila); **Serena De Pellegrin** (Padova); **Michelangelo Bartolo** (Bergamo), **Chiara Zucchella** (Verona); **Pietro Spinelli**, **Irene Aprile** (Roma); **Caterina Pistarini**, **Valeria Pingue** (Pavia); **Mirco Soda** (Bolzano).

## Ethics Statement

Comitato Etico Regione Toscana. Area Vasta Sud Est. Prot. 376/CEAVSE del 17-12-2015.

## Author Contributions

MM, ND, PZ, GA, AM, MC, MGC, GP, MC, and IC designed the experiment; all members of the Italian OCS group provided data, LA, FP, AD, and VV collected and analyzed the data; MM, PZ, LA, and FP wrote the paper; MM, ND, PZ, GA, and AM provided critical feedback and gave final approval of the version to be published.

## Conflict of Interest Statement

The authors declare that the research was conducted in the absence of any commercial or financial relationships that could be construed as a potential conflict of interest.
